# The Influence of Congenital Heart Defect Repair Through a Right Subaxillary Thoracotomy on Postoperative Pulmonary Function and Prognosis in Small Infants

**DOI:** 10.31083/RCM46570

**Published:** 2026-05-25

**Authors:** Xiaonan Wang, Xiang Li, Chen He, Huixian Li, Sheng Wang

**Affiliations:** ^1^Department of Anesthesiology, Beijing Anzhen Hospital, Capital Medical University, Beijing Institute of Heart, Lung and Blood Vessel Diseases, 100029 Beijing, China; ^2^Department of Anesthesiology, The First Hospital of Tsinghua University, 100016 Beijing, China

**Keywords:** infant, congenital heart defects, cardiac surgical procedure, respiratory insufficiency

## Abstract

**Background:**

The influence of repair of congenital heart defect (CHD) via a right subaxillary thoracotomy (RSAT) on postoperative pulmonary function and prognosis in small infants is a key consideration.

**Methods:**

Data were collected from infants who underwent ventricular septal defect (VSD) repair or VSD and atrial septal defect (ASD) between March 2020 and September 2024. Based on propensity score matching, 80 small infants (weight <5 kg and age <6 months) were selected, of which 40 underwent VSD repair or VSD, and ASD repair through an RSAT, while 40 underwent VSD repair or VSD and ASD repair through a standard median sternotomy (SMS). Perioperative respiratory parameters, morbidity, and mortality were compared to assess the influence of the RSAT approach on pulmonary function and postoperative outcomes in small infants.

**Results:**

Primary outcome: there were no significant differences (*p* > 0.05) in the perioperative oxygenation index and alveolar–arterial oxygen gradient between the two surgical approaches. No significant differences were also observed between the two groups in the other respiratory parameters, including peak airway pressure, partial pressure of oxygen, and partial pressure of carbon dioxide. The operating time (150 ± 20 min vs. 163 ± 28 min;* p *< 0.05) was shorter in the RSAT group compared to the SMS group. There were no deaths in either group. The complication rate was low in both groups, with no significant difference between the groups (*p* > 0.05).

**Conclusion:**

Compared with SMS, the RSAT approach for the repair of congenital heart defects does not increase the risk of postoperative respiratory insufficiency and yields comparable outcomes in low-weight small infants.

## 1. Introduction

Pulmonary insufficiency is relatively common following cardiac surgery [1]. Surgical trauma, cardiopulmonary bypass (CPB), and atelectasis caused by lung collapse during CPB all increase the incidence of pulmonary insufficiency. Pulmonary complications are of greater concern in infants, since the respiratory system of infants is incompletely developed and their number of alveoli is much fewer than in adults, which results in a more limited respiratory reserve. Stayer *et al*. [2] reported that age was the only significant factor affecting changes in both pulmonary dynamic compliance and total respiratory resistance, and therefore was a stronger predictor of changes in respiratory mechanics in infants undergoing heart surgery. Infants with congenital heart defect (CHD) often have pulmonary artery hypertension and pulmonary infection before surgery [2], which further complicates pulmonary function after surgery. In order to minimize surgical trauma, CHD repair through a right subaxillary thoracotomy (RSAT) has been increasingly adopted and has been proven to be safe and feasible. Its cosmetic effect and the avoidance of sternotomy-related complications have made it increasingly popular among patients. However, in order to facilitate visual exposure, compression of the right lung is inevitable in CHD repair through a right subaxillary thoracotomy, raising concerns about procedure-related pulmonary injury, particularly in small infants. Small infants weighing less than 5 kg and who are less than 6 months old may be more predisposed to lung injury using the right subaxillary approach for CHD repair. In small infants with smaller body size, compression of the non-dependent lung due to surgical manipulation is relatively more common compared to older children, which may cause tissue injury and trigger the release of inflammatory factors. Compression of the non-dependent lung results in over-inflation of the dependent lung which can cause pulmonary barotrauma of the dependent lung. Currently, clinical evidence regarding the effects of the right subaxillary approach on pulmonary function in low-weight small infants is limited.

Therefore, this study aimed to evaluate the influence of the right subaxillary thoracotomy for CHD repair on postoperative pulmonary function and postoperative morbidity and mortality in this vulnerable population.

## 2. Materials

A total of 198 infants who underwent ventricular septal defect (VSD) repair or combined VSD and atrial septal defect (ASD) repair performed by the same surgical team at our institution between March 2020 and September 2024 were retrospectively reviewed. In this group, 40 low-weight infants (weight <5 kg and age <6 months) who underwent surgery via the RSAT approach were selected for analysis. To minimize selection bias and balance prognostic factors, a 1:1 matched-pairs case–control design was used.

Each infant in the RSAT group was matched to one in the standard median sternotomy (SMS) group from the same year based on body weight (difference ≤0.5 kg), age (difference ≤0.5 months), defect size (difference ≤2 mm), patch usage, sex, and an identical operating surgeon and anesthesiologist. Inclusion criteria were: (1) pediatric patients diagnosed with an isolated VSD or VSD combined with ASD; (2) <5 kg and <6 months at the time of operation; (3) no obvious symptoms of cardiac insufficiency. Exclusion criteria: children on ventilation or other forms of assisted ventilation before surgery.

Exclusion criteria: Children requiring invasive ventilation or any form of assisted ventilation before surgery.

This study was approved by the ethics committee of the Beijing Anzhen Hospital, and because of the retrospective aspect of the study, the need for consent was waived.

### 2.1 Anesthesia Management 

After induction with intravenous ketamine, sufentanil, and rocuronium, tracheal intubation with a cuffed tube was performed. Midazolam, sufentanil, and rocuronium were used for maintenance of anesthesia. Mechanical ventilation was implemented using the Avance CS^2^ anesthesia machine (Avarice CS^2^, Datex-Ohmeda,3030 Ohmeda Drive, Madison, WI 53718, USA) with 50%–60% oxygen. Pressure-controlled ventilation was used in both groups, and peak inspiratory pressure was initially set at 17 cm of H_2_O and adjusted upwards or downwards so that a tidal volume of 8–10 mL/kg ideal body weight was reached. Respiratory rate was set at 24–26 breaths per minute with an inspiratory: expiratory ratio of 1:1 to maintain end tidal carbon dioxide of 35–40 mmHg. Ventilation was stopped during CPB. After cardiopulmonary bypass, manual ventilation of 3–5 consecutive respiratory cycles was performed with peak airway pressure at 30 cm H_2_O to prevent lung atelectasis.

### 2.2 Surgical Technique

The RSAT group: as described previously, after satisfactory anesthesia, the infants were placed in the left lateral decubitus position with the right side elevated about 45°–60°, and the infant’s right arm resting on the face. The thoracic cavity was entered through the fourth intercostal space. A wet gauze patch was placed between the right lung and the pericardium to protect the lung and expose the pericardium. The pericardium was opened longitudinally 1–2 cm anterior to the phrenic nerve, and was put on traction to assist in the exposure of the operative field. CPB was established through aortic, inferior, and superior vena cava cannulation, the ascending aorta was cross-clamped, and cardioplegia was achieved by infusing a cold crystalloid cardioplegic solution into the ascending aorta root, and the procedures were performed under mild hypothermia. The defects were closed using patches secured with a continuous suture technique. Inflate the lungs and squeeze the tissues to fully de-air through the left heart vent, the patient was rewarmed and CPB was discontinued gradually.

The SMS group: the infant was placed in the supine position, and the heart was exposed through a standard midline sternotomy. The surgical procedures used in the median sternotomy group were the same as those in the RSAT group.

### 2.3 Mechanical Ventilation and Extubation in the ICU

All patients were transferred to the Intensive Care Unit (ICU) under anesthesia after surgery, and ventilated using volume-controlled ventilation with a tidal volume of 8–10 mL/kg body weight on arrival in the ICU. The ventilator mode and parameters were adjusted according to the result of arterial blood gas analysis, and the timing of extubation was determined by the patients’ hemodynamic, respiratory, and neurological status.

### 2.4 Data Collection

Baseline and preoperative data were collected, including age, sex, height, weight, body surface area, pneumonia history, systolic pulmonary arterial pressure (PAP) (estimated by transthoracic echocardiography through the measurement of the peak velocity of the tricuspid regurgitation), and the size of the VSD.

Respiratory parameters including peak inspiratory pressure (PIP), arterial oxygen partial pressure (PaO_2_), arterial carbon dioxide partial pressure (PaCO_2_), oxygenation index (PaO_2_/FiO_2_), and alveolar-arterial oxygen gradients [P(A-a)O_2_] were collected at 4 time points: T1, after anesthesia induction; T2, the end of operation; T3, ICU admission and T4, before extubation. P(A-a)O_2_ was calculated using the formula: P(A-a)O_2_ = 713 × FiO_2_–PaO_2_–PaCO_2_.

Perioperative variables included: operation time, cardiopulmonary bypass time, aortic cross-clamp time, ventilation time, reintubation within the first 24 hours of extubation, use of any form of noninvasive ventilation support after extubation within the first 24 hours, ICU length of stay, duration of postoperative hospitalization, postoperative complications and mortality.

### 2.5 Statistical Analysis

Statistical analyses were performed using SPSS version 21.0 (IBM Corp., USA). A *p* value < 0.05 was considered statistically significant. Continuous variables were expressed as mean ± standard deviation (SD) or median (range) according to their distribution. Continuous data were compared using the *t*-test or Mann–Whitney U test, as appropriate, while categorical variables were analyzed using Pearson’s chi-square or Fisher’s exact test. Repeated measures of respiratory parameters were analyzed using repeated-measures ANOVA.

## 3. Results

### 3.1 Patient Characteristics

The demographic characteristics of both groups were similar, as shown in Table 1. The incidence of pneumonia was very low in both groups and was not significantly different between the groups (*p* > 0.05). The median systolic pulmonary arterial pressure was 64.50 (range from 51.25 to 78.75) mmHg in the RSAT group and 59.50 (range from 25.25 to 78.25) mmHg in the SMS group; *p* > 0.05. The median diameters of the VSD were the same in each group with a mean value of 10 (range from 10 to 12) mm. The mean CPB time, aortic cross-clamp time in the RSAT group and SMS group were (66 ± 13) min *vs* (65 ± 15) min (*p* > 0.05); (37 ± 9) min *vs* (35 ± 11) min (*p* > 0.05), respectively.

**Table 1.** **Baseline characteristics and intraoperative variables**.

Variable	Total	SMS group	RSAT group	*p*
	(n = 80)	(n = 40)	(n = 40)	
Age (month)	3.95 ± 1.00	3.92 ± 1.07	3.98 ± 1.04	0.873
Sex (m)	46	20	26	0.175
Weight (kg)	5.54 ± 0.63	5.44 ± 0.65	5.65 ± 0.59	0.149
Height (cm)	62.50 (60.00, 64.00)	63.00 (60.00, 64.00)	62.00 (60.00, 63.00)	0.597
Body surface area	0.44 (0.42, 0.45)	0.44 (0.42, 0.46)	0.44 (0.42, 0.45)	0.985
Pneumonia history	0.00 (0.00, 0.75)	0.00 (0.00, 0.00)	0.00 (0.00, 1.00)	0.460
PAP before surgery (mmHg)	63.00 (27.75, 78.75)	59.50 (25.25, 78.25)	64.50 (51.25, 78.75)	0.314
VSD size (mm)	10.00 (10.00, 12.00)	10.00 (10.00, 12.00)	10.00 (10.00, 12.00)	0.814
Operating time (min)	180 (180, 180)	163 ± 28	150 ± 20	0.039
CPB time (min)	72.21 ± 14.15	65 ± 15	66 ± 13	0.846
Aortic cross-clamp time (min)	40.28 ± 10.24	35 ± 11	37 ± 9	0.535

Data are presented as mean ± standard deviation (SD), median (interquartile range). PAP, pulmonary artery pressure; VSD, ventricular septal defect; CPB, cardio-pulmonary bypass; SMS, standard median sternotomy.

### 3.2 Primary Outcome 

**Fig. 1.**
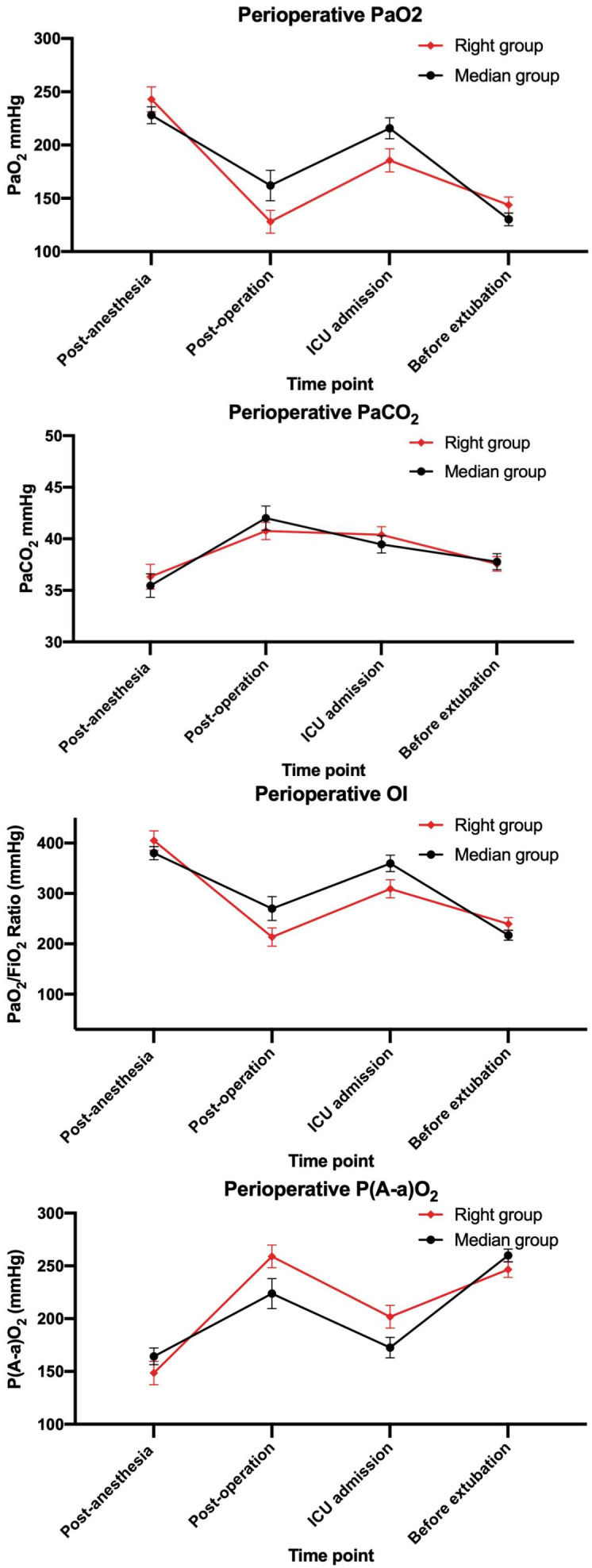
**Perioperative changes of PaO_2_, PaCO_2_, OI, and P(A-a)O_2_**. OI, oxygenation index; PaO_2_, arterial oxygen partial pressure; PaCO_2_, arterial carbon dioxide partial pressure; P(A-a)O_2_, alveolar-arterial oxygen tension gradient.

No statistically significant differences were detected between the RSAT and SMS groups in perioperative respiratory parameters, including PIP, PaO_2_, PaCO_2_, oxygenation index (PaO_2_/FiO_2_), and alveolar–arterial oxygen gradient [P(A–a)O_2_] (Table 2 and Fig. 1). Although both groups exhibited a trend toward decreased PaO_2_ and PaO_2_/FiO_2_ and increased P(A–a)O_2_ at the end of surgery, these variations were comparable between groups and did not reach statistical significance after adjustment for multiple comparisons. Although isolated differences in PaO_2_/FiO_2_ and P(A–a)O_2_ reached statistical significance at T2 and T3, the effect sizes were modest, all values remained within clinically unremarkable ranges, and these findings were not consistent across time points or adjusted for multiple comparisons. Therefore, they do not support a clinically meaningful difference in postoperative pulmonary function.

**Table 2.** **Perioperative respiratory parameters at four time points (T1–T4)**.

Variable	Time	SMS group (mean ± SD)	RSAT group (mean ± SD)
PIP (cmH_2_O)	T1	17.00 ± 1.11	16.88 ± 1.24
	T2	16.75 ± 1.51	16.80 ± 1.24
	T3	16.98 ± 1.79	16.48 ± 1.66
	T4	16.55 ± 1.75	16.65 ± 1.39
PaO_2_ (mmHg)	T1	228.02 ± 50.32	242.94 ± 73.41
	T2	162.05 ± 89.92	128.06 ± 68.09
	T3	215.75 ± 61.69	185.58 ± 68.79
	T4	130.17 ± 37.28	143.72 ± 47.22
PaCO_2_ (mmHg)	T1	35.46 ± 7.21	36.33 ± 7.58
	T2	42.00 ± 7.39	40.76 ± 5.27
	T3	39.45 ± 5.26	40.39 ± 4.96
	T4	37.78 ± 4.90	37.57 ± 4.46
PaO_2_/FiO_2_	T1	380.03 ± 83.86	404.89 ± 122.34
	T2	270.08 ± 149.86	213.43 ± 113.49
	T3	359.58 ± 102.81	309.30 ± 114.65
	T4	216.95 ± 62.13	239.53 ± 78.70
P(A-a)O_2_	T1	164.32 ± 49.77	148.53 ± 70.05
	T2	223.75 ± 89.62	258.99 ± 67.82
	T3	172.59 ± 61.44	201.83 ± 67.84
	T4	259.86 ± 38.80	246.52 ± 47.39

T1, after anesthesia induction; T2, the end of operation; T3, ICU admission; T4, before extubation; PIP, peak inspiratory pressure; SMS, standard median sternotomy; SD, standard deviation; RSAT, right subaxillary thoracotomy.

### 3.3 Secondary Outcome 

The median duration of mechanical ventilation, ICU stay, and postoperative hospitalization were comparable between the RSAT and SMS groups: ventilation time 22.0 h (range 19.25–44.50) vs 24.0 h (range 22.0–46.0), *p* > 0.05; ICU stay 3.0 days (range 1.25–4.0) vs 3.0 days (range 2.0–5.0), *p* > 0.05; postoperative hospital stay 6.0 days (range 6.0–10.0) vs 7.5 days (range 6.0–11.0), *p* > 0.05.

No reintubation was required in both groups. Two infants needed noninvasive ventilation support within 24 hours after extubation in the SMS group. Complications were rare in both groups: one right-sided pneumothorax occurred in the RSAT group; and one re-exploration for pericardial effusion and one neurologic complication occurred in the SMS group.

## 4. Discussion

Surgical repair of CHD through a right subaxillary thoracotomy has been performed for more than 20 years. It has been welcomed by patients and their families with its excellent cosmetic results, and its efficacy and safety have been established in several clinical studies [3, 4, 5]. Nonetheless, careful patient selection is required for this approach. In adults, because of the depth of the thoracic cavity, exposure of the surgical field and surgical manipulation can be difficult. Therefore, some medical centers consider patients with a body weight of more than 30 kg or a Body Mass Index (BMI) >30 kg/m^2^ are not suitable for the right subaxillary thoracotomy approach [5]. In children with a shallow chest, the poor operative exposure and limited space for surgical manipulation can be a significant issue. Therefore, the use of RSAT in children under 15 kg has not been recommended in earlier studies due to the difficulty in aorta cannulation [6]. With increasing experience, in recent years, the RSAT has been applied to both toddlers and infants. However, this approach, which involves exposing the heart by retracting the right lung, has caused concerns about postoperative respiratory dysfunction in infants. In this study, we found that the right subaxillary approach for correction of congenital heart defect did not increase respiratory dysfunction in low-weight small infants when compared with the conventional median sternotomy approach.

Perioperative pulmonary dysfunction in pediatric patients with CHD is very common and can be related to several factors: (1) pulmonary insufficiency intrinsic to the underlying CHD. The abnormal changes of cardiac structure in congenital heart disease and the resultant abnormal hemodynamics, especially the increase of pulmonary blood flow, are one of the chief mechanisms for perioperative respiratory dysfunction [7, 8]. The left-to-right shunt pathophysiology in VSDs leads to an increase in pulmonary blood flow and a reduction in pulmonary compliance [9]. Surgical correction can reverse the excessive pulmonary blood flow and rapidly improve the mechanical properties of the lungs. (2) Pulmonary insufficiency associated with cardiac surgery. In addition to surgical trauma, CPB used in cardiac surgery triggers a systemic inflammatory response [10, 11, 12, 13] and can also induce an ischemia-reperfusion injury of the lung [14, 15]. During the right subaxillary thoracotomy approach for correction of CHD, lateral thoracotomy induced changes in ventilation physiology may be another factor contributing to postoperative pulmonary insufficiency. In the right subaxillary thoracotomy approach, in order to adequately expose the surgical field, the right lung is often significantly compressed and one-lung ventilation is instituted. As a result, the right lung experiences more shear injury during re-expansion, while the left lung is predisposed to barotrauma from large tidal volumes, especially in small infants whose lung compliance is limited [16]. Theoretically, lung injury can be more severe following a right subaxillary thoracotomy than in a median sternotomy. An *et al*. [17] summarized their work with correction of congenital heart defects via a right subaxillary thoracotomy in 836 pediatric patients (median age 3.5 years, median weight 13.6 kg). They found that preservation of pulmonary function was vital, and suggested applying lower tidal mechanical ventilation volumes with positive expiratory pressure [17]. In our study, the postoperative PaO_2_/FiO_2_ ratio values were reduced to the range of acute lung injury (even less than 200 mmHg) and the P(A-a)O_2_ was elevated in both groups, which indicated the presence of lung injury during the surgery. However, the reduction in PaO_2_/FiO_2_ and the P(A-a)O_2_ elevation were comparable (without statistical difference) between the RSAT group and the SMS group at different postoperative time points, which indicated that the extra one-lung compression of the right subaxillary thoracotomy approach did not cause more damage to the lung in low-weight small infants. Since the size of the VSD, pulmonary vascular resistance, and history of pneumonia in both groups were comparable, the pulmonary insufficiency of the patients was mainly attributed to the surgical procedure.

A previous retrospective study [4] showed that the incidence of postoperative lung atelectasis was 3.88% in the right subaxillary thoracotomy approach for CHD in patients aged 9.6 months to 17 years old who weighed 7.5 to 58 kg, but recovered before discharge. In our study, no lung atelectasis occurred in either group. Other clinical pulmonary outcomes defined as mechanical ventilation time, number of patients in need of noninvasive ventilation support, and the incidence of pulmonary complications were also similar between the two groups which confirmed the safety of the right subaxillary thoracotomy for CHD regarding postoperative pulmonary function in low-weight small infants.

Other prognostic outcomes in Table 3 such as ICU stay, postoperative hospital stay, and postoperative complications were also similar between the two groups. These findings are consistent with previous studies showing that the right subaxillary thoracotomy approach is a good alternative to the traditional mid-sternotomy approach in low-weight small infants.

**Table 3.** **Prognostic variables**.

Variables	Total	Median group	Right group	*p*-value
Ventilation time (h)	24.00 (21.00, 46.00)	24.00 (22.00, 46.00)	22.00 (19.25, 44.50)	0.218
ICU stay length (day)	3.00 (2.00, 4.75)	3.00 (2.00, 5.00)	3.00 (1.25, 4.00)	0.122
Postoperative hospitalization (day)	7.00 (6.00, 10.00)	7.50 (6.00, 11.00)	6.00 (6.00, 10.00)	0.109
Complications	5	4	1	0.170

Data are presented as median (interquartile range). ICU, intensive care unit. Complications are listed as absolute numbers.

### Limitations

The current study has certain limitations, including potential biases caused by the retrospective design. The use of matched-pair analysis maximized the similarity of the baseline characteristics in the two groups, which at least partly makes up for the insufficiency of the retrospective design. In the current study, the degree of lung injury was investigated from clinical perspectives, which included clinical outcomes and gas exchange parameters, but lacked serum biochemical assays, which may more accurately reflect the extent of the lung injury.

## 5. Conclusion

Compared with the conventional median sternotomy, the right subaxillary thoracotomy approach for CHD repair does not exacerbate postoperative respiratory insufficiency in low-weight small infants and yields comparable short-term prognostic outcomes. RSAT can therefore be considered a safe and effective alternative to median sternotomy for CHD surgery in this population, with the additional benefit of a more cosmetic incision.

## Availability of Data and Materials

Due to the laboratory’s policy or confidentiality agreements, we are unable to provide the raw data. If you have any specific questions about the data, we will do our best to provide more detailed explanations and clarifications.
